# Transcatheter Aortic Valve Implantation and Cognitive Function: Treating the Heart, Altering the Brain?

**DOI:** 10.1016/j.shj.2025.100695

**Published:** 2025-08-05

**Authors:** Nikolaos Pyrpyris, Kyriakos Dimitriadis, Panagiotis Papanagiotou, Konstantinos Tsioufis

**Affiliations:** First Department of Cardiology, School of Medicine, National and Kapodistrian University of Athens, Hippokration General Hospital, Athens, Greece; Department of Radiology, Aretaieion University Hospital, School of Medicine, National and Kapodistrian University of Athens, Athens, Greece; First Department of Cardiology, School of Medicine, National and Kapodistrian University of Athens, Hippokration General Hospital, Athens, Greece

Dear Editor:

We read with interest the study by Potluri et al.,^1^ evaluating alterations in cognitive function of patients with baseline mild cognitive impairment undergoing transcatheter aortic valve implantation (TAVI). The authors showed that at 6 months follow-up, the Mini Montreal Cognitive Assessment score significantly increased in almost 90% of patients, with a mean increase of 2.3 points. This study, besides documenting the safety of TAVI in patients with mild cognitive decline, also suggests a benefit of the intervention in cognitive function, therefore raising several clinical implications.

The known risk for postprocedural stroke and transient ischemic attacks after TAVI has questioned its effect in cognitive function, while the physiology behind potential improvements remains unknown. In this setting, the restoration of physiologic hemodynamics post-TAVI and increase of cardiac output could be related with enhanced cerebral blood flow. Recently the CArdiac outPut, cerebral blood flow and cognition In patients with severe aortic valve stenosis undergoing Transcatheter Aortic valve implantation (CAPITA) study,^2^ that evaluated post-TAVI cognitive scores along with magnetic resonance imaging, found an improvement of cognitive function in 32% of patients. However, despite cerebral blood flow also increased at 3 months follow-up, no significant correlation of cognitive improvement with cerebral blood flow changes was reported. It is possible that the overall enhancement of patient health status after the intervention may help toward improving some aspects of cognitive test scores. However, as cerebral blood flow changes after TAVI have not been well evaluated, further studies should investigate the effect of TAVI in cerebral hemodynamics, along with cognitive function, aiming to better understand the mechanisms explaining the observed benefit.

Moreover, the investigators reported a sex-specific benefit of TAVI, with greater improvements in the Mini Montreal Cognitive Assessment score in female patients. Females, despite having a quicker cognitive decline, also exhibit greater cognitive reserve than males, thus having greater baseline scores.^3^ Considering this, the finding of Potluri et al.^1^ comes in contrast to similar studies, where patients with the worst cognitive status at baseline experienced greater benefit.^2^ Given the well-documented differences in both epidemiology of dementia and cerebral hemodynamics between sexes,^3^^,^^4^ future research in larger samples should evaluate if there is a sex-related effect while exploring the pathogenetic links behind this association.

Notably, similar mechanisms of cognitive improvement through restoration of cerebral perfusion have been described after carotid artery stenting, with improvements primarily observed in processing speed and executive control functions. This parallel supports the hypothesis that improving cerebral hemodynamics—whether through cardiac or extracranial vascular interventions—may underlie cognitive benefit in selected patient populations.^5^

Finally, despite a benefit in cognitive function may be present in a substantial number of patients, some will still experience cognitive decline. This decline may be associated with the acute, subclinical infarcts due to periprocedural factors (rapid pacing, embolization), identified as new-onset or increase in white matter hyperintensity at follow-up magnetic resonance imaging.^2^ Unfortunately, no risk factors for this clinically significant, particularly for older individuals, outcome have been established. Therefore, besides documenting the positive effect of TAVI in cognitive function, it is also crucial to identify risk factors for cognitive decline and evaluate preventive strategies in high-risk individuals.

## Funding

The authors have no funding to report.

## Disclosure Statement

The authors report no conflict of interest.
